# Diagnostic performance and clinical impact of blood metagenomic next-generation sequencing in ICU patients suspected monomicrobial and polymicrobial bloodstream infections

**DOI:** 10.3389/fcimb.2023.1192931

**Published:** 2023-06-26

**Authors:** Qilong Liu, Xiaojing Liu, Bingxue Hu, Huan Xu, Rongqing Sun, Pengfei Li, Yunwei Zhang, Hongfu Yang, Ning Ma, Xiaoge Sun

**Affiliations:** ^1^ Intensive Care Unit, The First Affiliated Hospital of Zhengzhou University, Zhengzhou, Henan Province, China; ^2^ Department of Neurology, The First Affiliated Hospital of Zhengzhou University, Zhengzhou, Henan Province, China; ^3^ Department of Scientific Affairs, Vision Medicals for Infectious Diseases, Guangzhou, Guangdong Province, China

**Keywords:** bloodstream infection, intensive care unit, blood culture, metagenomics next generation sequencing, polymicrobial infection

## Abstract

**Introduction:**

Early and effective application of antimicrobial medication has been evidenced to improve outcomes of patients with bloodstream infection (BSI). However, conventional microbiological tests (CMTs) have a number of limitations that hamper a rapid diagnosis.

**Methods:**

We retrospectively collected 162 cases suspected BSI from intensive care unit with blood metagenomics next-generation sequencing (mNGS) results, to comparatively evaluate the diagnostic performance and the clinical impact on antibiotics usage of mNGS.

**Results and discussion:**

Results showed that compared with blood culture, mNGS detected a greater number of pathogens, especially for *Aspergillus spp*, and yielded a significantly higher positive rate. With the final clinical diagnosis as the standard, the sensitivity of mNGS (excluding viruses) was 58.06%, significantly higher than that of blood culture (34.68%, *P*<0.001). Combing blood mNGS and culture results, the sensitivity improved to 72.58%. Forty-six patients had infected by mixed pathogens, among which *Klebsiella pneumoniae* and *Acinetobacter baumannii* contributed most. Compared to monomicrobial, cases with polymicrobial BSI exhibited dramatically higher level of SOFA, AST, hospitalized mortality and 90-day mortality (*P*<0.05). A total of 101 patients underwent antibiotics adjustment, among which 85 were adjusted according to microbiological results, including 45 cases based on the mNGS results (40 cases escalation and 5 cases de-escalation) and 32 cases on blood culture. Collectively, for patients suspected BSI in critical condition, mNGS results can provide valuable diagnostic information and contribute to the optimizing of antibiotic treatment. Combining conventional tests with mNGS may significantly improve the detection rate for pathogens and optimize antibiotic treatment in critically ill patients with BSI.

## 1 Introduction

The bloodstream infection (BSI) is a potentially lethal complication that frequently results in multi-organ failure, septic shock, and disseminated intravascular coagulation. It also has a high mortality rate and large socioeconomic costs. The annual incidence of BSI can be as high as 204/100, 000 and the fatality rate 20.6%, accounting for about 10% intensive care unit (ICU) inpatients (Goto and Al-Hasan, 2013; Martinez and Wolk, 2016). It is widely accepted that the length of stay in patients of BSI is long, and any delay in the treatment may result in increased mortality (Ferrer et al., 2014). Therefore, the rapid and appropriate antibiotics treatment is now recognized as a key measure in the care of patients suspected BSI.

Blood culture is currently the standard method for diagnosing BSI. Other conventional microbiological tests (CMTs), such as direct microscopic examination (DME), nucleic acid amplification tests (NAAT), and serological tests, can also be applied in detecting pathogens. However, the practical use of culture and DME is constrained by time-consuming and low detection rates (Li et al., 2019). And NAAT and serological tests can only detect a few suspected pathogens. Recently, metagenomic next-generation sequencing (mNGS) have shown great potential in pathogen detection for patients suspected BSI (Geng et al., 2021; Hu et al., 2021; Jing et al., 2021). Thousands of pathogens are known to infect humans, but only a fraction of them can be identified using current clinical microbiology methods. The newly developed mNGS technology enables the rapid diagnosis of unexplained infections (Yan et al., 2021). Due to its nearly full coverage of causative pathogens and short turnaround time, mNGS has become a reliable method for the early identification of BSI. However, only a few studies have focused on the application of mNGS in ICU patients with BSI, and the clinical impact of mNGS is unclear (Geng et al., 2021; Hu et al., 2021; Yan et al., 2021; He et al., 2022). Therefore, this study retrospectively analyzed the clinical data of 162 critically BSI patients who received blood mNGS diagnoses to explore the clinical value and applicability.

## 2 Materials and methods

### 2.1 Study design

From July 1, 2019 to August 31, 2022, 219 patients suspected BSI were admitted to the ICU of the First Affiliated Hospital of Zhengzhou University. This single-center study retrospectively analyzed the clinical data of these patients, including general demographic information, clinical characteristics and outcomes. The inclusion criteria included: (1) age ≥ 18 years, (2) met the diagnostic criteria of sepsis 3.0 (Singer et al., 2016) and suspected BSI, (3) expected ICU hospitalization time ≥ 24 hours. Exclusion criteria included: (1) only received blood culture or mNGS test; (2) died before the results of blood culture or mNGS were returned; (3) repeated tests during 14 days after the first mNGS test. This study was approved by the Ethics Committee of the First Affiliated Hospital of Zhengzhou University (approval number: 2023-KY-0069).

### 2.2 Blood sample preparation

Disposable sterile needles were used to collect 25ml of whole blood samples within 24 hours of inclusion, among which, 20ml for blood culture (two bottles of aerobic and anaerobic), and 5ml for mNGS detection within 8 hours.

### 2.3 Blood culture

The BACT/ALERT 3D automatic bacterial culture instrument was applied for testing the blood samples. After incubating the samples for 5-7 days, the positive results were judged by at least two professional physicians to make sure the report is accurate. The final clinical positive result was concluded when any blood culture reported positive results, and environmental microbial contamination was excluded. If common bacteria found on the skin (such *Staphylococcus epidermidis*, *Propionibacterium acnes*, *Clostridium*, and *Corynebacterium diphtheriae*) or in the environment (like *Acinetobacter.spp*, *Bacillus.spp)* thrive in the oxygen bottle, the majority of these bacteria are considered contamination, however they may be considered as pathogenic in the following conditions: a) The same microorganism was cultured from blood samples collected from different body parts; b) The same microorganism has been isolated multiple times, and the results of drug susceptibility are the same.

### 2.4 Blood mNGS test and data analysis

Patients’ blood samples of 3-4 mL were collected, put in EDTA tubes, and kept at room temperature for 3-5 minutes before the plasma was separated and centrifuged for 10 min at 1,600 g at 4°C within 8 hours after collection. Using the TIANamp Micro DNA Kit (DP316, TIANGEN BIOTECH, Beijing, China), DNA was extracted from 300µL plasma according to the manufacturer’s instructions. Human DNA was removed using Benzonase (Qiagen) and Tween20 (Sigma) (Amar et al., 2021). The extracted DNA specimens were used for the constructing DNA libraries through DNA fragmentation, end repair, adapter ligation and PCR amplification. Subsequently, using single reads of 75 bp on the Illumina NextSeq 550 (Illumina, San Diego, California, USA) technology, libraries with proven quality were sequenced (Ren et al., 2018). For negative controls, PBMC samples with 10^5^ cells/mL from healthy donors in parallel with each batch were also prepared (Deng et al., 2022; Tao et al., 2022), using the same protocol, and sterile deionized water was extracted alongside the specimens to serve as non-template controls (NTC) (Miller et al., 2019).

For bioinformatics analyses, trimmomatic (Bolger et al., 2014) was used to remove low quality reads, adapter contamination, and duplicate reads, as well as those shorter than 50 bp. Low complexity reads were removed by Kcomplexity with default parameters (Bolger et al., 2014). Human sequence data were identified and excluded by mapping to a human reference genome (hg38) using Burrows-Wheeler Aligner software (Li and Durbin, 2009). The remaining sequencing information was aligned to the most recent databases for bacteria, viruses, fungi, and protozoa (NCBI; ftp:/ftp.ncbi.nlm.nih.gov/genomes). Reads that met the criteria for being considered unique were those with alignment lengths greater than 80%, sequence identities greater than 90%, and suboptimal to optimal alignment score ratios lower than 0.8. The reads per million (RPM) ratio, or RPM-r, was defined as the RPM_sample_/RPM_NTC_ (the RPM corresponding to a specific species or genus in the clinical sample divided by the RPM in the NTC), and a positive detection was reported for a certain species or genus if the RPM-r was ≥10.

### 2.5 Criteria for a positive mNGS result

The specifically mapped read number (SMRN) of each microbial taxonomy was normalized to SMRN per 20 million (M) of total sequencing reads (SDSMRN, standardized SMRN). The criteria for reporting mNGS result as followings (Qin et al., 2021):

- SDSMRN ≥3 (mycobacteria excluded) for bacteria
- SDSMRN ≥3 for fungi/DNA virus
- SDSMRN ≥1 for RNA virus
- SDSMRN ≥100 for parasites
- SDSMRN ≥3 for *Mycoplasma/Chlamydia* spp.
- SDSMRN ≥1 (or SDSMRNG ≥1 at genus level) for *Mycobacterium tuberculosis* (MTB) complex
- Reported Nocardia spp. by mNGS all considered positive

### 2.6 The diagnostic performance analysis

To compare diagnostic performance between mNGS and blood culture, a composite final diagnosis was retrospectively made by comprehensively considering the results of various examinations (laboratory findings, mNGS results and imagological findings), the patient’s clinical manifestations and response to treatment. Then, the final clinical diagnosis was applied as reference standard, and the sensitivity, specificity, positive predictive value (PPV) and negative predictive value (NPV) were calculated (Blauwkamp et al., 2019).

### 2.7 Statistical analysis

SPSS 25.0 (IBM) and GraphPad Prism 9.3 (GraphPad Software) were utilized for statistical analysis and figures drawing. Continuous variables were presented as medians and interquartile value (IQR, P25, P75), categorical variables were expressed as counts and percentages. Comparison of categorical variables was done with chi-square test. The Mann-Whitney U test was employed for comparing the differences in the continuous variables. *P* < 0.05 was considered to indicate statistical significance.

## 3 Results

### 3.1 Patient characteristics

**Figure 1:**
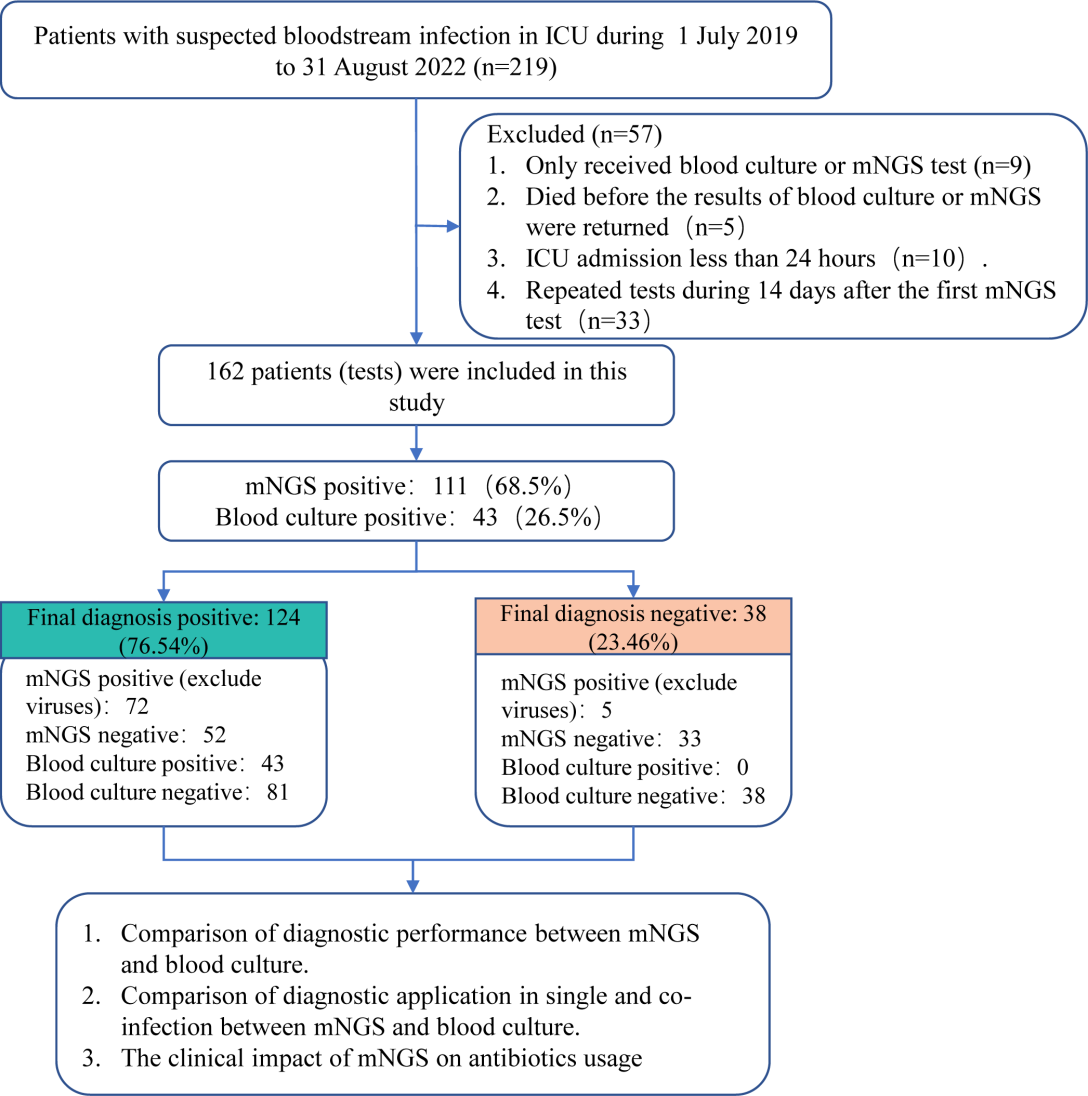
A schematic of the study profile.

A total of 162 patients suspected BSI were finally included in this study (**Figure 1**). As shown in **Table 1**, 57.41% (93/162) patients had underlying diseases, such as hypertension (29.63%), diabetes (15.43%), and cerebrovascular disease (11.11%). The vast majority of patients (91.36%) had complications during hospitalization, mainly including respiratory system disease (55.56%), cardiovascular disease (48.77%) and renal insufficiency (25.31%). The most common source of infection was the lung (37.04%), followed by the abdomen (17.9%). A total of 124 (76.5%) patients were finally diagnosed as BSI, among them 78 were infected with single pathogens and the remaining 46 were co-infection. **Table 2** demonstrated the clinical data of the patients. The median APACH II and SOFA were 17 and 10, respectively. A total of 45.68% and 58.64% cases died at 28-day and 90-day admission, respectively.

**Table 1:** Demographic characteristic of patients.

Clinical features	n=162
Gender, male	99 (61.1)
Age, years	55 (44, 67)
**Underlying diseases, n (%)**	94 (58.02)
Hypertension	48 (29.63)
Diabetes mellitus	25 (15.43)
Cerebrovascular disease	18 (11.11)
Chronic cardiac disease	17 (10.49)
Chronic respiratory diseases	7 (4.32)
Chronic liver disease	7 (4.32)
Malignancy	5 (3.09)
Chronic kidney disease	4 (2.47)
Other	13 (8.02)
**Comorbidity, n (%)**	150 (92.59)
Disease of respiratory system	90 (55.56)
Disease of cardiovascular system	79 (48.77)
Renal insufficiency	41 (25.31)
Nervous system disease	19 (11.73)
Disease of digestive system	10 (6.17)
Immunosuppression	9 (5.56)
Hypohepatia	7 (4.32)
Anemia	3 (1.85)
Other	12 (7.41)
**Infectious sources, n (%)**	n=162
Lung	60 (37.04)
Abdomen	29 (17.9)
Gastrointestinal tract	13 (8.02)
Urinary tract	13 (8.02)
Catheter-related	12 (7.41)
Skin and soft tissue	10 (6.17)
Biliary tract	9 (5.56)
Primary	6 (3.7)
Central Nervous System	4 (2.47)
Other	6 (3.7)
**Infectious type, n (%)**	125 (77.16)
single infection	78 (62.9)
coinfection	46 (37.1)

**Table 2:** Clinical characteristic of patients.

Clinical features	Number
APACHE II	17 (12, 22)
SOFA	10 (7, 15)
Time to progress to sepsis (days)	3 (2, 6)
WBC (×10^9^/L)	12.58 (8.25, 19.71)
Neutrophil (%)	87.55 (81.65, 92.5)
Platelet (×10^9^/L)	101 (42, 196.75)
ALT (U/L)	39.5 (17, 88.25)
AST (U/L)	44 (20, 95.5)
Total bilirubin(μmol/L)	18.52 (9.7, 35.28)
DBil (µmol/L)	9.65 (5.42, 25)
Serum albumin (g/L)	30.05 (25.53, 34.53)
D-dimer (mg/L)	2.74 (1.07, 5.94)
Prothrombin time (s)	13.8 (12.5, 16.33)
CRP (mg/L)	102.13 (44.2, 172)
PCT (ng/mL)	5.06 (0.96, 24)
Urea nitrogen (mmol/L)	12.25 (7.5, 19)
Creatinine (µmol/L)	94 (61, 170.25)
Mortality (28 days)	74 (45.68)
Mortality (hospitalization)	7 (4.32)
Mortality (90 days)	95 (58.64)
Duration of ICU stay (days)	12.5 (6, 22.25)
Total hospitalization time (days)	17.5 (10, 37.25)
Mechanical ventilation time (hours)	68.5 (0, 230.5)
Vasoactive drug use time (hours)	45.5 (0, 135.15)
Total hospitalization cost (CNY)	199246.92 (92737.73, 402597.59)

### 3.2 Comparison of mNGS and blood culture for pathogen detection

**Figure 2:**
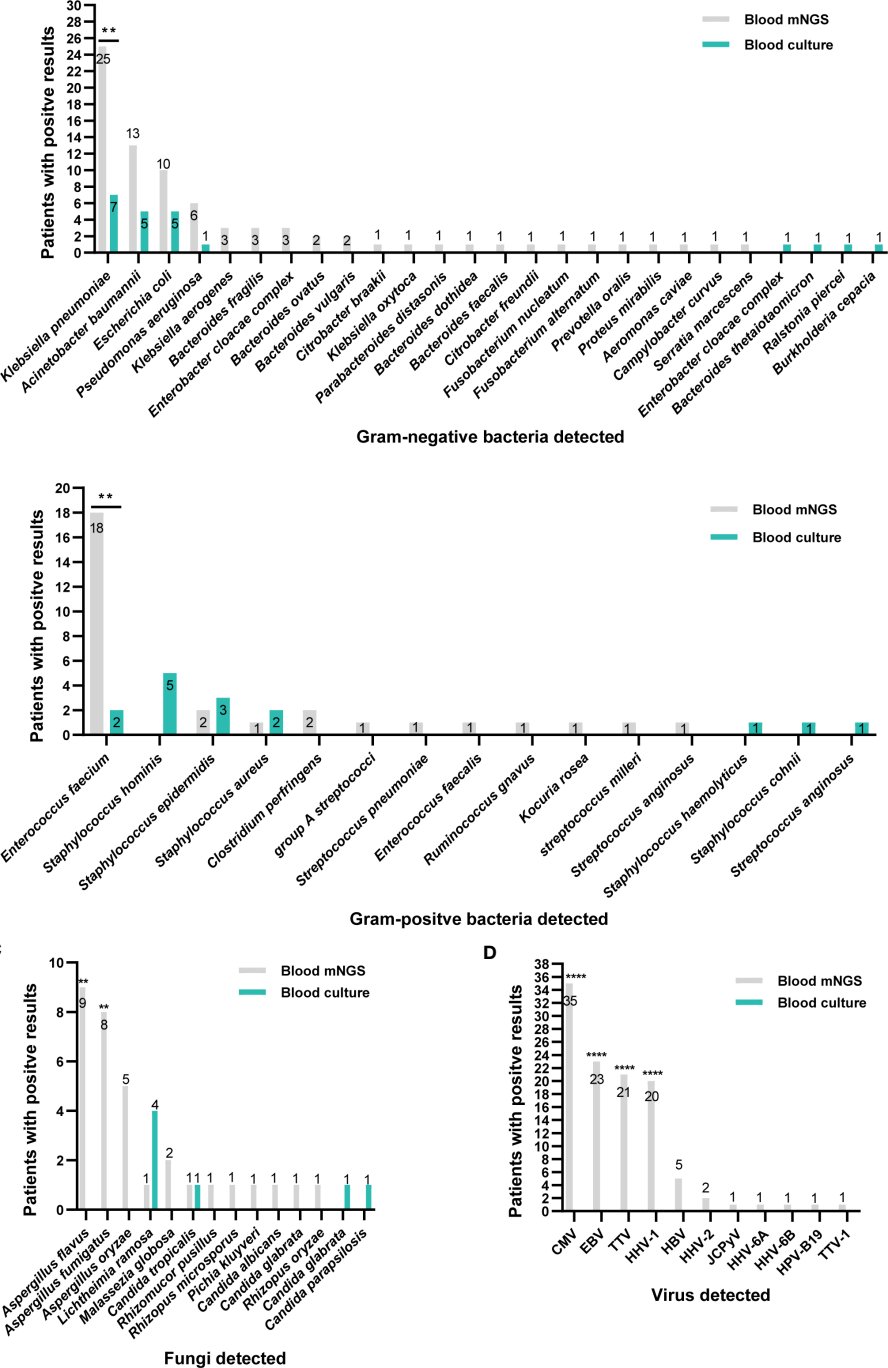
Distribution of pathogens identified by blood mNGS and blood culture. **(A)** Gram-negative bacteria. **(B)** Gram-positive bacteria. **(C)** fungi. **(D)** Viruses. **P<0.01 and ****P<0.0001. CMV, Cytomegalovirus; EBV, Epstein-Barr virus; TTV, Torque Teno virus; HHV, human herpes virus; HBV, Hepatitis B virus; JCPyV, JC polyomavirus.

Firstly, we analyzed the ability of blood culture and blood mNGS for pathogens detection. As shown in **Figure 2**, a total of 66 pathogenic species were detected in 124 patients by blood culture and mNGS, including Gram-positive bacteria, Gram-negative bacteria, fungi, and viruses. Generally, blood mNGS detected a greater variety of microbes than culture. *Klebsiella pneumoniae*, *Acinetobacter baumannii*, and *Enterococcus faecium* were the most common bacteria. In terms of fungal detection by mNGS, *Aspergillus flavus*, *Aspergillus fumigatus*, and *Aspergillus oryzae* were most frequently detected and they were only detected by mNGS. Compared with blood culture, mNGS could detect a greater number of pathogens (253 vs 34, *P*<0.0001). Among them, the detection ability of mNGS for *Klebsiella pneumoniae*, *Enterococcus faecium*, *Aspergillus flavus* and *Aspergillus fumigatus* was significantly stronger than blood culture (*P*<0.05, **Figures 2A-C**). In 127 cases with prior antibiotics administration, mNGS had a significantly higher detection rate on *Klebsiella pneumoniae* and *Enterococcus faecium* than blood culture; but in 35 cases without prior antibiotics, no significant difference was observed between mNGS and blood culture in detecting *Klebsiella pneumoniae* (**Supplementary Figure 1**).

Moreover, the main viruses detected by mNGS were *Human betaherpes virus 5* (CMV), *Human gamaherpesvirus 4* (EBV) and *Torque teno virus* (TTV). mNGS made up for the shortcomings of the inability to detect viruses in blood culture (**Figure 2D**).

### 3.3 Comparison of clinical diagnostic value in BSI between mNGS and blood culture

**Figure 3:**
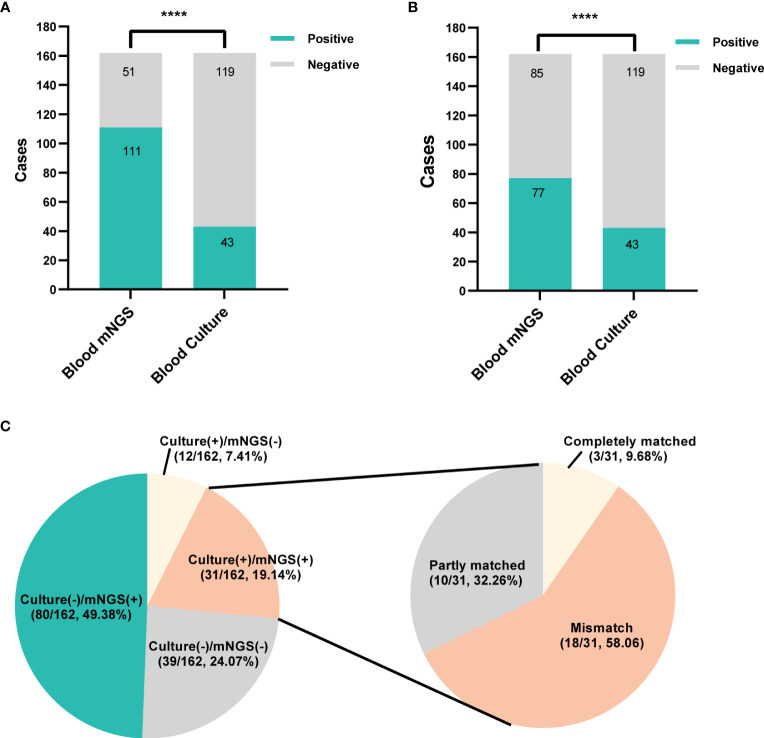
The positivity distribution of blood mNGS and blood culture. **(A)** The positive rates of mNGS and blood culture for all cases (n=162). ****P<0.0001. **(B)** The positive rates of mNGS and blood culture for all cases (n=162) after viruses detected by mNGS were excluded. ****P<0.0001. **(C)** Pie chart demonstrating the positivity distribution of mNGS and blood culture for all tests. The double-positive samples were further categorized as completely matched, partly matched (at least one overlap of pathogens was observed) and mismatched.

Among 162 patients, mNGS showed positive result in 111 cases, with a positive rate of 68.5%, which was significantly higher than blood culture (26.5%, 43/162, *P*<0.0001, **Figure 3A**). After virus removal, the positive rate for mNGS was 47.5% (77/162), which was still significantly higher than that for blood culture (*P*<0.0001, **Figure 3B**). Blood culture and mNGS showed double positive results in 31 (19.14%) patients, among which the positive pathogens were partly matched in 32.26% (10/31) case and completely matched in 9.68% (3/31) cases (**Figure 3C**).

**Figure 4:**
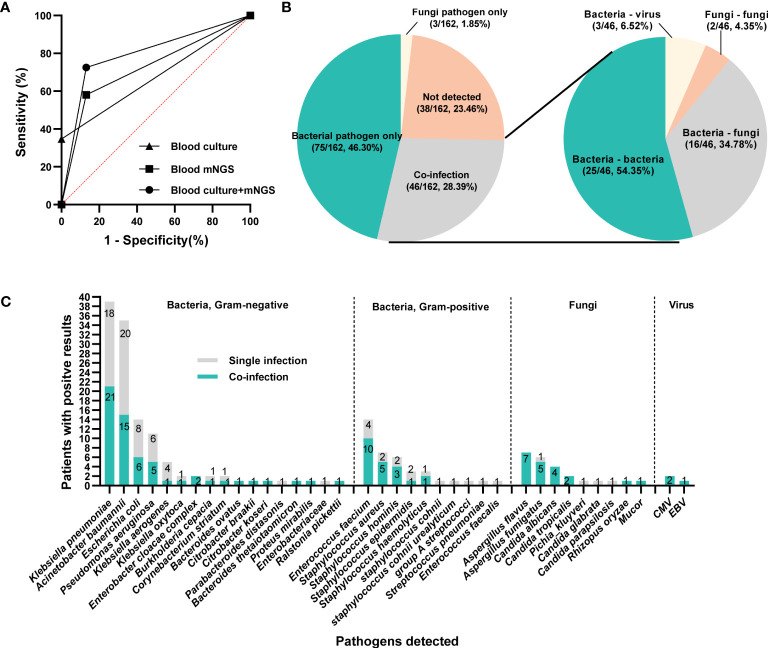
The infection types and responsible pathogens. **(A)** ROC curve of mNGS, blood culture and mNGS+culture with reference to final clinical diagnosis. **(B)** Pie chart demonstrating the infectious type, and the co-infection was further categorized as bacteria-bacteria, bacteria-fungi, bacteria-virus and fungi-fungi. **(C)** the distribution of microbial species that involving in single infection and co-infection.

Under the circumstances of excluding viruses and using blood culture as standard, the sensitivity, specificity, PPV and NPV of mNGS were 58.14%, 56.3%, 32.47% and 78.82%, respectively, (**Table 3**). Still, using final clinical diagnosis as standard (**Table 4**), the sensitivity of mNGS was 58.06%, significantly higher than that of blood culture (34.68%, *P*<0.001). The specificity, PPV and NPV between mNGS and blood culture showed no statistical difference. And combining the results of blood mNGS and culture, the sensitivity, specificity, PPV and NPV were 72.58%, 86.84%, 94.74% and 49.25%, respectively. Additionally, the AUC for mNGS, blood culture, and blood mNGS plus culture, were 0.7245 (95% CI: 0.589 to 0.758), 0.6734 (95% CI: 0.639 to 0.810) and 0.7971 (0.719 to 0.876), respectively (**Figure 4A**).

**Table 3:** The diagnostic performance of mNGS in ICU patients with BSI as compared to blood culture.

	Blood culture positive	Blood culture negative	Sensitivity (%)	Specificity (%)	PPV (%)	NPV (%)	AUC	Youden index (%)
mNGS positive	25	52	58.14	56.3	32.47	78.82	0.57	14.44
mNGS negative	18	67						

**Table 4:** Comparison of diagnostic performance between mNGS and blood culture in ICU patients with BSI.

	Final clinical diagnosis positive	Final clinical diagnosis negative	Sensitivity (%)	Specificity (%)	PPV (%)	NPV (%)	AUC (95% CI)	Youden index (%)
mNGS positive	72	5	58.06	86.84	93.51	38.82	0.7245 (0.639-0.810)	44.9
mNGS negative	52	33						
Blood culture positive	43	0	34.68	100	100	31.93	0.6734 (0.589-0.758)	34.68
Blood culture negative	81	38						
mNGS+blood culture positive	90	5	72.58	86.84	94.74	49.25	0.7971 (0.719-0.876)	59.42
mNGS+blood culture negative	35	32						

### 3.4 Comparison of diagnostic application in single and mixed infections between mNGS and blood culture

According to the microbiological results (**Figure 4B**), 38 cases (23.46%) were regarded as non-infection, 75 cases (46.30%) as single bacterial infection in which 3 patients (1.85%) were identified as single fungi infection, and the remaining 46 cases (28.40%) mixed pathogens (≥2 organisms) infection. Among these 46 patients with co-infection (**Figure 4B**), 25 cases (54.35%) were infected with multiple bacteria, 16 cases (34.78%) with bacteria and fungi, and 2 cases with multiple fungi (one infected with *Candida tropicalis*, *Aspergillus flavus* and *Rhizopus oryzae*, the other infected with *Aspergillus flavus* and *Aspergillus fumigatus*), and 3 cases with bacteria and virus. In terms of specific microorganism species, *Klebsiella pneumoniae* and *Acinetobacter baumannii* contributed to the majority cases of co-infection, followed by *Enterococcus faecium* and *Aspergillus flavus* (**Figure 4C**). Meanwhile, BSI caused by *Aspergillus* spp. and *Candida* spp. were more likely to be associated with polymicrobial infections (**Figure 4C**). Comparison of clinical characteristics between mixed and single infections showed that cases with mixed infections exhibited significantly higher level of SOFA, AST, hospitalized mortality and 90-day mortality (*P*<0.05, **Table 5** & **Supplementary Figure 2**).

**Table 5:** Comparison of clinical characteristics between mixed and single infections.

Characteristics	Single infection (n=78)	Mixed infection (n=46)	*P* value
Gender, male,	47 (60.26)	32 (69.57)	0.298
Age, years	57 (46, 69.25)	51 (38.5, 60.75)	0.041
With underlying diseases	41 (52.56)	25 (54.35)	0.848
Presenting comorbidity	69 (88.46)	44 (95.65)	0.174
APACHE II	15.5 (11, 21)	17 (11.75, 24.5)	0.211
SOFA	9 (7, 13)	13 (8.75, 17.25)	0.013
Time to progress to sepsis (days)	3 (2, 6.25)	3 (1.75, 5.25)	0.486
WBC (×10^9^/L)	12.14 (7.75, 18.82)	14.15 (9.83, 21.55)	0.218
Neutrophil (%)	87.45 (81.95, 92.55)	89.5 (79.53, 92.83)	0.822
Platelet (×10^9^/L)	92.5 (42.75, 232.75)	103 (33.5, 174.75)	0.485
ALT (U/L)	30 (15.75, 73.75)	51.5 (22.5, 98.75)	0.076
AST (U/L)	34.5 (18, 80)	71 (28.5, 148)	0.003
Total bilirubin (μmol/L)	16.72 (8.95, 30.9)	21.6 (14.37, 42.3)	0.057
DBil (µmol/L)	8.8 (5.05, 18.9)	11.37 (7.85, 30.63)	0.067
Serum albumin (g/L)	30.05 (24.85, 35.45)	29.9 (25.33, 33.48)	0.578
D-dimer (mg/L)	2.74 (1.01, 5.74)	2.1 (1.11, 4.34)	0.508
Prothrombin time (s)	13.75 (12.2, 15.73)	13.9 (12.88, 19.43)	0.090
CRP (mg/L)	105.5 (48.95, 196.22)	111.3 (44.06, 172.17)	0.727
PCT (ng/mL)	4.35 (1.14, 28)	8.48 (0.83, 28.53)	0.798
Urea nitrogen (mmol/L)	13.15 (8.08, 18.93)	11.7 (6.23, 25.28)	0.741
Creatinine (µmol/L)	94 (59.85, 148.75)	103.5 (61, 251.5)	0.687
Mortality (28 days)	33 (42.31)	23 (50)	0.406
Mortality (hospitalization)	1 (1.28)	5 (10.87)	0.016
Mortality (90 days)	41 (52.56)	33 (71.74)	0.035
Duration of ICU stay (days)	14 (6, 25.75)	12.5 (6, 24.25)	0.444
Total hospitalization time (d)	22.5 (11, 44.75)	15.5 (8, 39.25)	0.266
Mechanical ventilation time (h)	54.5 (0, 238)	128.5 (14.25, 275.5)	0.142
Vasoactive drug use time (h)	36.6 (0, 123.5)	46.5 (3, 144)	0.750
Total hospitalization cost (CNY)	208799 (72110.99, 448181.77)	206138.14 (132801.84, 432240.25)	0.690

*Klebsiella pneumoniae* contributed to 19 and 29 cases of single infection and co-infection, respectively (**Supplementary Table 1**). The majority of the infectious sources for single and mixed *Klebsiella pneumoniae* infection was lung. Except higher SOFA in mixed infection group (*P*=0.031), none other significant differences were observed in demographic and clinical characteristics between single and mixed *Klebsiella pneumoniae* infection group. A total of 17 and 16 patients were infected with single and mixed *Acinetobacter baumannii*, respectively. Compared with *Acinetobacter baumannii* single infection, co-infected patients exhibited lower serum albumin and urea nitrogen (*P*=0.04, **Table 6**).

**Table 6:** The characteristics of cases received antibiotics de-escalation according to mNGS result.

Case No.	1	2	3	4	5
Gender	Female	Male	Female	Male	Male
Age (years)	48	35	67	50	41
Underlying diseases	Hypertension, Chronic kidney disease	/	Cerebrovascular disease, Chronic cardiac disease	Chronic liver disease	Hypertension
Comorbidity	/	Disease of respiratory system, Disease of cardiovascular system	/	Disease of respiratory system	Acute cerebral infarction
Infectious sources	Catheter-related	Lung	Urinary tract	Lung	Catheter-related
Blood Culture	Negative	Negative	*Candida glabrata*	Negative	*Acinetobacter baumannii*
Other CMTs	Sputum culture (negative)	Smear (negative), sputum culture (*Klebsiella pneumoniae*, *Pseudomonas aeruginosa*)	None	BALF culture (*Klebsiella pneumoniae*)	None
mNGS	*Escherichia coli*	*Pseudomonas aeruginosa*	*Human gamaherpesvirus 4 (EBV), Candida glabrata*	*Klebsiella pneumoniae, Human betaherpesvirus 5 (CMV), Human alphaherpesvirus 1, Torque teno virus, Torque teno virus 1, Human gamaherpesvirus 4 (EBV)*	*Acinetobacter baumannii, Human betaherpesvirus 5 (CMV), Torque teno virus 5, Lichtheimia ramosa*
Final confirmed pathogens	*Escherichia coli*	*Pseudomonas aeruginosa*	*Candida glabrata*	*Klebsiella pneumoniae*	*Acinetobacter baumannii*
Disease severity					
APACHE II	10	21	6	16	10
SOFA	8	10	8	9	6
Time to progress to sepsis (days)	2	3	2	5	11
Mechanical ventilation time (hours)	0	308	0	168	159
Vasoactive drug use time (hours)	0	258	70	202	13
Duration of ICU stay (days)	8	56	32	14	22
Total hospitalization time (days)	28	93	45	48	22
Laboratory findings					
WBC (×10^9^/L)	3.11	2.99	5.97	7.27	10.62
Neutrophil (%)	87.2	61.62	82.0	93.1	96.4
CRP (mg/L)	199	137.2	122	150	202
PCT (ng/mL)	100	0.32	0.933	8.33	16
Treatments					
Empirical treatment	Meropenem, Vancomycin	Rifamycin, Biapenem, Polymyxin	Cefepime, Tigacycline	Imipenem, Carpofungin, Tiraconine	Meropenem, Tegacycline, Teicolanin
Treatment after Final confirmed pathogen diagnosis	Meropenem	Rifamycin, Amtriannan	Voriconazole	Ceftazidime avibactam, Carpofungin, Tiraconine	Meropenem, Polymyxin
The basis for antibiotics adjustment	mNGS	mNGS + sputum culture	mNGS + blood culture	mNGS + BALF culture	mNGS + blood culture
Outcomes					
28 days death	Survival	Survival	Survival	Survival	Death
Hospital death	Survival	Survival	Survival	Survival	Survival
90 days death	Survival	Survival	Survival	Survival	Death

### 3.5 The clinical impact of mNGS on antibiotics usage

**Figure 5:**
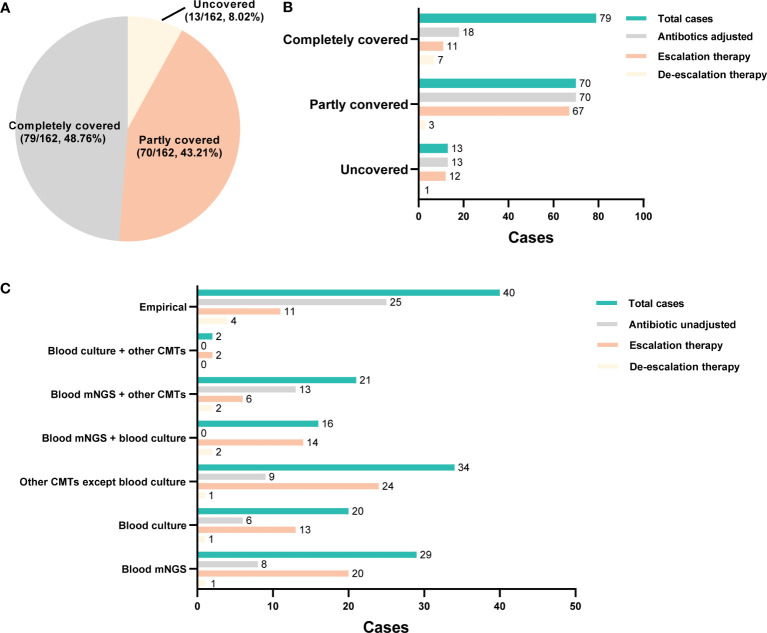
The adjustment of empirical antimicrobial treatment. **(A)** Pie chart illustrating the coverage of initial empirical antibiotics usage. **(B)** the adjustment of empirical antibiotics usage in each group. **(C)** the basis for the adjustment of empirical antibiotics usage.

The initial empirical treatment completely covered all identified pathogens in 48.76% (79/162) cases, partially covered in 43.21% (70/162) cases, and not covered any pathogen in 8.02% (13/162) cases (**Figure 5A**). The adjustment of empirical treatment strategy is made by summarizing microbiological results, laboratory findings and the response to the initial treatment. In this study, 101 patients underwent adjustment of antibiotics usage, including 18 cases [11 cases escalation and 7 cases de-escalation (antibiotics were discontinued or changed to a narrower spectrum)] in the complete coverage group, 70 cases (67 cases escalation and 3 cases de-escalation) in the partly coverage group, and 13 cases (12 cases escalation and 1 case de-escalation) in the uncovered group (**Figure 5B**). The adjustment basis of antibiotics usage was illustrated in **Figure 5C**. Antibiotics were adjusted based on clinical experience in 15 patients (15/101, 14.85%), including 11 cases escalation and 4 cases de-escalation.

According to microbiological results, 86 patients (86/101, 85.15%) received antibiotics adjustment. Among them, 14 cases were adjusted based only on the blood culture results (13 cases escalation and 1 case de-escalation), 16 patients were adjusted according to blood mNGS plus culture results (14 cases escalation and 2 cases de-escalation), and 8 cases were adjusted based on blood mNGS plus other CMT results (6 cases escalation and 2 cases de-escalation). Moreover, among 21 cases whose adjustment basis was only on blood mNGS, 20 cases resulted in an escalation of treatment and 1 case de-escalation of treatment. Totally, the antibiotics usage of 45 and 32 cases were adjusted according to mNGS and blood culture results, respectively (**Figure 5C**). In addition, mNGS and blood culture results contributed to the confirmation of empirical treatment in 21 cases and 6 cases, respectively (**Figure 5C**). Totally, among 101 patients received antibiotics adjustment, mNGS played a role in 45 patients, and the remaining 56 patients were adjusted according to other microbiological results; there was no statistical difference in mortality (28 days, hospitalization, 90 days), duration of ICU stay, total hospitalization time, mechanical ventilation time, vasoactive drug use time, and total hospitalization cost between the two groups (*P*>0.05, **Supplementary Table 3**).

### 3.6 Antibiotics de-escalation according to mNGS result

A total of 5 cases received antibiotics de-escalation based on mNGS result (**Table 6**). The infectious sources of 2 cases were lung, and the other 2 catheter, the remaining 1 urinary tract. mNGS detected at least 1 pathogen for all 5 cases, whereas only 2 cases were shown to be pathogens positive by blood culture (Case 3 *Candida glabrata*, Case 5 *Acinetobacter baumannii*). The final definite diagnosis of all 5 cases were made depending on mNGS results with CMTs. Among them, the confirmation of causative pathogen and the antibiotics adjustment of Case 1 was made only based on mNGS result. Case 1 was complicated with uremia, renal insufficiency, and high fever. Blood PCT was 100 ng/ml and CRP 199 mg/L, indicating critical infection. First, Meropenem and vancomycin were empirical prescribed. Then, according to *Escherichia coli* reported by mNGS, vancomycin was stopped and meropenem continued for anti-infection. Finally, case 1 discharged with recovered condition. Case 2-5 were also diagnosed as single pathogen infection. Case 3 was diagnosed as the infection of *Candida glabrata* based on the results of blood mNGS and culture, cefepime and tigacycline was replaced by voriconazole. Similarly, in Case 2, 4 and 5, empirical antibiotics were discontinued or changed to a narrower spectrum according to the results of blood mNGS and culture. Except Case 5, 4 cases achieved clinical cure or improvement. Case 5 was diagnosed as catheter-related *Acinetobacter baumannii* infection depending on the results of blood mNGS and culture, meropenem + tegacycline + teicolanin was replaced by meropenem + polymyxin. Although his initial disease severity was relative mild (APACHE II 10, SOFA 6), he developed acute cerebral infarction and died after abandoning treatments.

## 4 Discussion

The present study retrospectively compared the diagnostic performance of blood mNGS and culture in ICU patients suspected BSI, and results indicated that mNGS exhibited stronger potential in diagnosing BSI than blood culture, as evidenced by detecting a larger number of pathogens and yielding a higher sensitivity. In addition, mNGS could provide effective etiological basis for the adjustment of antibiotics usage.

BSI is a serious systemic infectious disease caused by the invasion of pathogenic microorganisms, which is a frequent complication in the ICU, predisposing patients to septic shock and death. According to studies conducted on septic patients within the first six hours of recorded hypotension, every hour delay in effective antibiotics usage resulted in an average 7.6% drop in survival rates, and if appropriate therapy was not administered within 24 hours, the survival probability for severe sepsis dropped from 80 to 10% (Kumar et al., 2006; Ferrer et al., 2014). Therefore, timely and accurate detection of pathogenic microorganisms is crucial. Blood culture is considered the gold standard for diagnosing BSI, but it is time-consuming and lower sensitivity (10% to 30%) (Previsdomini et al., 2012; Yealy et al., 2014). Serum immunological tests and PCR techniques can only detect a few specific pathogens and require specialized kits and primers.

In recent years, mNGS, as high-throughput sequencing, has been continually improved and popularized, providing a new and powerful means for pathogen diagnosis. mNGS offers rapid and unbiased testing, characterized by broad-range pathogen detection, which can fulfil the limitations of culture. It has been widely accepted that mNGS has obvious advantages in detecting a broad-spectrum of pathogens, especially for the diagnosis of *Mycobacterium tuberculosis*, virus, anaerobic bacteria and fungi (Gu et al., 2019; Duan et al., 2021). In this study, we showed that blood mNGS not only detected a greater variety of microbes than culture, but also exhibited advantage in detecting common pathogens including *Klebsiella pneumoniae* and *Enterococcus faecium*. Notably, *Aspergillus flavus*, *Aspergillus fumigatus*, and *Aspergillus oryzae* were only detected by mNGS. Therefore, mNGS provided a good complementary to blood culture to maximize the overall detection of *Aspergillus* spp. Furthermore, in 18 cases, both culture and mNGS were positive but their results were not always consistent, this may be due to a number of variables, including the type of pathogen, the usage of antibiotics, and the mNGS reporting threshold. The prior application of antibiotics will significantly reduce the detection sensitivity of blood culture, but has less impact on the sensitivity of mNGS (Miao et al., 2018), so mNGS will additionally detect the virus in situations where it is difficult to culture the virus. For example, in this study, the *Klebsiella pneumoniae* detection rate of mNGS was superior to that of culture (16.54% vs 3.15%, *P <*0.001) in cases with, but not without, antibiotic exposure. Besides, false negative results for mNGS may occur for samples with positive blood cultures because the pathogen load is below the mNGS detection limit or because the sample has a high host load, which impairs the detection.

mNGS has shown great potential in the diagnosis of clinical infectious diseases such as respiratory tract infections (Zheng et al., 2021; Diao et al., 2022), BSI (Eichenberger et al., 2022) and central nervous system infections (Frémond et al., 2015; Chiu et al., 2017). Emerging studies have demonstrated that mNGS exhibited superiority than culture test in identifying infectious diseases (Miao et al., 2018; Qian et al., 2020; Chen et al., 2021). Herein, mNGS exerted a positive rate of 68.5%, and reduced positive rate of 47.5% after virus removal, both were significantly higher than culture (26.5%, *P*<0.0001). The research of XIE et al (Xie et al., 2021) reported that for patients with pulmonary infection, the positive rate of pathogen detection by mNGS was 82.14%, which was significantly better than the 35.71% of conventional methods. In terms of ability for differentiating infection from non-infection, a study by TAO et al (Tao et al., 2022) found that for the diagnosis of infectious diseases, the sensitivity of mNGS was 74.32%, while the sensitivity of conventional detection was 38.2%. Similar results were also reflected in another study, which reported that the sensitivity of mNGS in diagnosing infectious diseases was 67.40%, which was better than culture (23.60%) (Duan et al., 2021). Consistently, our results showed that the sensitivity of mNGS was superior to that of culture (58.06% vs 34.68%, *P*<0.001), similar to a previous study performed on patients with suspected infectious disease (52.5% vs 34.2%; P < 0.01) (Miao et al., 2018). The high sensitivity of mNGS may be due to the longer survival time of pathogenic DNA and the little effect of antibiotics on mNGS results. Combined with previous studies, the results of this study further confirmed that the sensitivity of mNGS in diagnosing infectious diseases is significantly higher than traditional etiological detection. In this study, the PPV and NPV of mNGS in diagnosing BSI were 93.51% and 38.82%, respectively. The lower NPV than previous studies (88.6% (Jing et al., 2021), 83.87% (Chen et al., 2021)) may be due to the fact that the final diagnosis of certain mNGS-negative cases was made based on the positive CMT results from bronchoalveolar lavage fluid (BALF), sputum and drainage fluid. Also, the majority of patients had antibiotic treatment before the mNGS test, which could have affected the NPV of the mNGS.

According to the largest series reported, polymicrobial BSI accounts for 6%-34% of BSIs (Kiani et al., 1979; Lin et al., 2010). In this study, 37.1% (46/124) BSI cases were polymicrobial infections. The relatively high rate may be explained by the critical conditions of ICU patients we enrolled. Moreover, mNGS showed superiority in contributing to the identification of co-infection than blood culture. mNGS contributed to the identification of co-infection in 32 cases, while blood culture played a role in 16 cases’ co-infection diagnosis. Mixed infections can hardly be identified by traditional culture methods, because various microorganisms interact with, and inhibit, each other (Semenec et al., 2023). Polymicrobial BSI is generally associated with a higher morbidity and more severe prognosis than monomicrobial BSI (Bouza et al., 2013). It was reported that mortality rate of hospitalized patients with polymicrobial BSI ranged from 21% to 63%, approximately twice the rate of those with monomicrobial infections (Cooper et al., 1990; Lin et al., 2010). Consistently, our results indicated that cases with mixed infection not only exhibited more serious condition (higher SOFA) but also showed a significant higher hospitalization mortality and 90-day mortality.

According to current recommendations, broad-spectrum antibiotic medication should be started as soon as feasible, ideally within one hour of the diagnosis of sepsis or septic shock, for the best therapeutic effectiveness (Evans et al., 2021). In actual clinical practice, however, it was discovered that roughly 46% of empirical antibiotic treatments were ineffective, increasing mortality rates by 35%, and roughly 50% of empirical antibiotic treatments were either unnecessary or broad-spectrum antibiotics, increasing the risk of antibiotic resistance and toxicity (Campion and Scully, 2018). Consequently, early antimicrobial therapy guidance, greater antibiotic stewardship, and improved clinical outcomes are all made possible by rapid and precise pathogens diagnosis for BSI. In the current study, initial empirical treatment completely covered all identified pathogens in 48.76% cases, and 62.35% cases underwent adjustment of antibiotics usage after diagnosis, among which, more cases were adjusted according to mNGS results than blood culture. For example, a patient who had previous pulmonary tuberculosis and presented with high fever, was diagnosed as septic shock on admission, with APACHE II 24 and PCT 7.1ng/ml. Prior to the return of microbiological results, empirical anti-infection treatment of imipenem was prescribed. Although the results of sputum culture and blood culture were negative, mNGS results suggested *Acinetobacter baumannii* infection, thus polymyxin combined with anti-infective therapy was added. Thereafter, the patient’s infection was controlled and was successfully transferred out of the ICU. The proportion of antibiotics adjusted based only on mNGS results was 21 cases, including 20 cases of escalation and 1 case of de-escalation adjustment. mNGS results guided the de-escalated antibiotics management in a total of 5 cases through helping to exclude active infection which allowed for antibiotic de-escalation. Furthermore, in 21 cases where broad-spectrum antibiotics are empirically used before detection, mNGS result can be used alone or as an effective supplement to CMTs for continuing the empirical treatment.

There were some deficiencies in this study. First, this research is a retrospective study, lacking the support of data from multi-center and large number of samples. Second, the majority of microorganisms discovered by mNGS have not been verified by molecular tests. Finally, the majority of patients underwent treatment prior to the mNGS or culture test, which may have impacted the sensitivity of culture and mNGS.

Taken together, our findings indicated that mNGS has great potential in the precise diagnosis and anti-infection treatment of clinical infectious diseases. Although it cannot replace blood culture detection technology, it can be used as a supplement to provide stronger diagnostic capabilities for BSI and optimize treatment.

## Data availability statement

The data presented in the study are deposited in the Genome Warehouse in the National Genomics Data Center (https://ngdc.cncb.ac.cn) repository, accession number PRJCA016199.

## Ethics statement

The studies involving human participants were reviewed and approved by Ethics Committee of the First Affiliated Hospital of Zhengzhou University (approval number: 2023-KY-0069). Written informed consent for participation was not required for this study in accordance with the national legislation and the institutional requirements.

## Author contributions

QL and XJ contributed to research design and paper writing. RS, PL, YZ, HY, NM and XS contributed to data collection. HX, BX, QL and XJ performed data analysis. QL and XJ revised the manuscript. All authors contributed to the article and approved the submitted version.
