# Supplementary material for: Diagnostic performance and clinical impact of blood metagenomic next-generation sequencing in ICU patients suspected monomicrobial and polymicrobial bloodstream infections
Source: Front Cell Infect Microbiol. 2023 Jun 26;13:1192931. doi: 10.3389/fcimb.2023.1192931 (PMC10330723; doi:10.3389/fcimb.2023.1192931)
Supplement: Supplementary file 1 [file DataSheet_1.docx]

Supplementary Material

Diagnostic performance and clinical impact of blood metagenomic next-generation sequencing (mNGS) in ICU patients suspected monomicrobial and polymicrobial bloodstream infections

**Qilong Liu^1†*^, Xiaojing Liu^2†^, Bingxue Hu^3^, Huan Xu^3^, Rongqing Sun^1^, Pengfei Li^1^, Yunwei Zhang^1^, Hongfu Yang^1^, Ning Ma^1^, Xiaoge Sun^1^**

**^*^Correspondence:**

Qilong Liu, Email: [13676936391@126.com](mailto:13676936391@126.com).


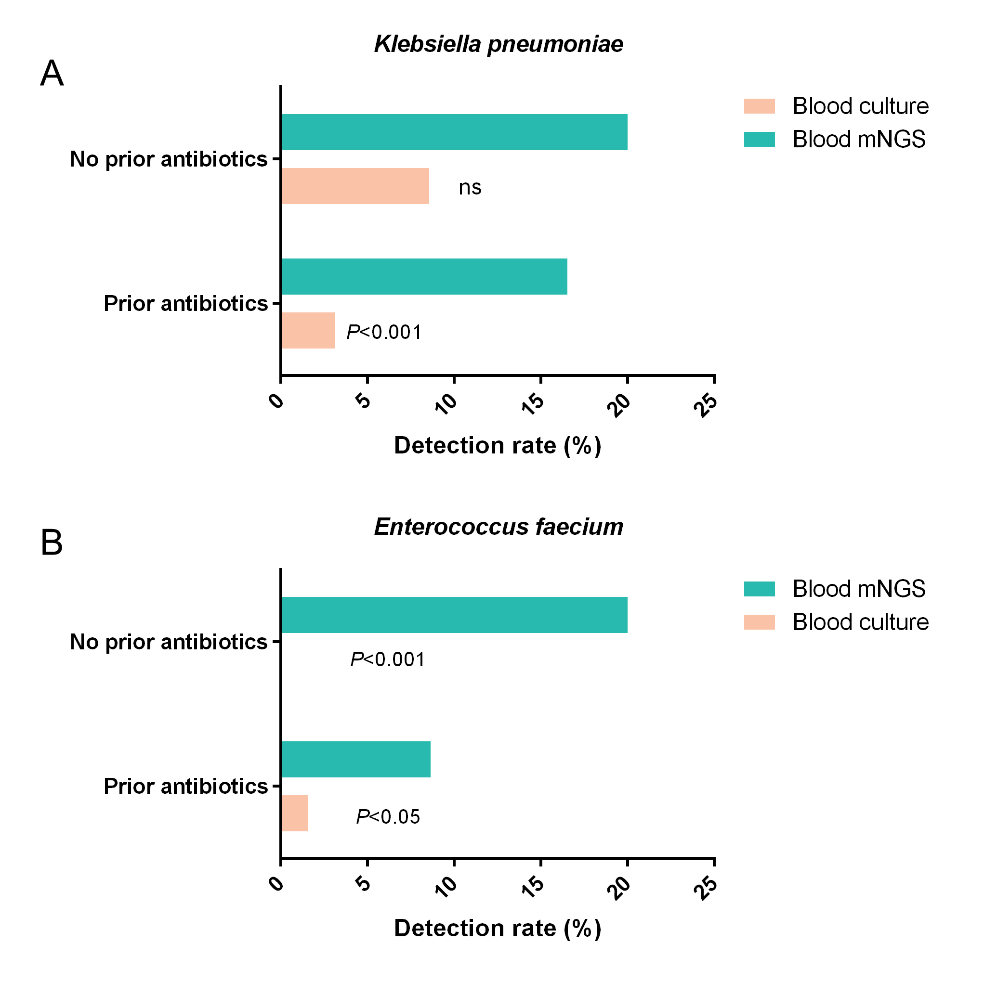


**Supplementary Figure 1.** Comparison of the detection rate of *Klebsiella pneumoniae* (A) and *Enterococcus faecium* (B) between blood culture and mNGS in cases with prior antibiotic administration or not.


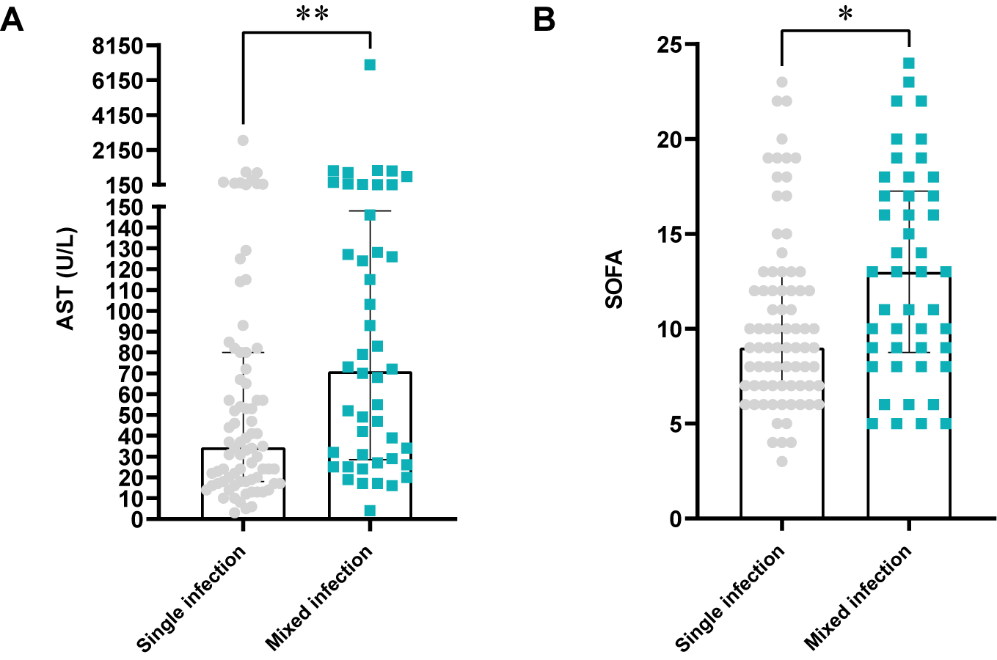


**Supplementary Figure 2.** Comparison of AST and SOFA between patients with mixed and single infections. The box-plot demonstrating the distribution of AST (A) and SOFA (B) in cases with single and mixed infection. **P*<0.05 and ***P*<0.01.

**Supplementary Table 1. Comparison of clinical characteristics between mixed and single *Klebsiella pneumoniae* infection**

| Characteristics | Single infection (n=19) | Mixed infection (n=19) | *P* value |
| --- | --- | --- | --- |
| Gender, male | 14 (73.68) | 14 (73.68) | 1.000 |
| Age, years | 57 (50, 68) | 50 (33, 70) | 0.129 |
| With underlying diseases | 7 (36.84) | 11 (57.89) | 0.194 |
| Presenting comorbidity | 16 (84.21) | 19 (100) | 0.071 |
| APACHE II | 15 (12, 22) | 22 (11, 26) | 0.474 |
| SOFA | 8 (6, 10) | 11 (9, 14) | **0.031** |
| Time to progress to sepsis (days) | 3 (2, 6) | 3 (2, 5) | 0.813 |
| WBC (×10^9^/L) | 10.55 (6.9, 15.92) | 11.8 (6.04, 14.78) | 0.737 |
| Neutrophil (%) | 86.6 (81.4, 93) | 86 (78.2, 93.9) | 0.988 |
| Platelet (×10^9^/L) | 76 (32, 194) | 120 (24, 192) | 0.530 |
| ALT (U/L) | 66 (13, 89) | 53 (17, 107) | 0.965 |
| AST (U/L) | 41 (18, 80) | 55 (26, 115) | 0.170 |
| Total bilirubin(μmol/L) | 20.3 (12.5, 34.7) | 19.1 (14, 36.1) | 0.827 |
| DBil (µmol/L) | 14.35 (7.8, 27.5) | 10.13 (7.9, 27.7) | 0.849 |
| Serum albumin (g/L) | 27.2 (24.1, 35.4) | 30.3 (26.3, 34.5) | 0.483 |
| D-dimer (mg/L) | 1.8 (1.03, 4.66) | 2.15 (0.6, 5.12) | 0.630 |
| Prothrombin time (s) | 13.8 (12.7, 16.1) | 14.4 (13.2, 22.2) | 0.321 |
| CRP (mg/L) | 153.53 (76, 328) | 96.67 (56.44, 176.97) | 0.184 |
| PCT (ng/mL) | 7.44 (1.5, 17.7) | 5.3 (0.74, 23) | 0.895 |
| Urea nitrogen (mmol/L) | 12.1 (10.3, 18.9) | 10.4 (5.1, 19.3) | 0.493 |
| Creatinine (µmol/L) | 81 (60, 157) | 89 (51, 248) | 0.953 |
| Mortality (28 days) | 9 (47.37) | 9 (47.37) | 1.000 |
| Mortality (hospitalization) | 0 (0) | 2 (10.53) | 0.146 |
| Mortality (90 days) | 10 (52.63) | 12 (63.16) | 0.511 |
| Duration of ICU stay (d) | 16 (7, 31) | 11 (6, 25) | 0.188 |
| Total hospitalization time (d) | 23 (7, 49) | 20 (13, 43) | 0.651 |
| Mechanical ventilation time (h) | 100 (20, 480) | 137 (1, 266) | 0.942 |
| Vasoactive drug use time (h) | 50 (24, 172.2) | 48 (10, 144) | 0.473 |
| Total hospitalization cost (CNY) | 285765.27 (88722.99, 535380) | 207277 (167127, 459072.62) | 0.693 |

**Supplementary Table 2. Comparison of clinical characteristics between mixed and single *Acinetobacter baumannii* infection**

| Characteristics | Single infection (n=17) | Mixed infection (n=16) | *P* value |
| --- | --- | --- | --- |
| Gender, male | 11 (64.71) | 12 (75) | 0.520 |
| Age, years | 60 (45, 69.5) | 51.5 (44, 58) | 0.149 |
| With underlying diseases | 7 (41.18) | 9 (56.25) | 0.387 |
| Presenting comorbidity | 14 (82.35) | 15 (93.75) | 0.316 |
| APACHE II | 19 (14, 22.5) | 17.5 (9, 22.75) | 0.745 |
| SOFA | 9 (7, 13.5) | 14.5 (8.25, 19) | 0.053 |
| Time to progress to sepsis (days) | 3 (1.5, 7.5) | 2.5 (1.25, 4.75) | 0.324 |
| WBC (×10^9^/L) | 13.91 (10.96, 18.64) | 12.65 (9.58, 16.54) | 0.377 |
| Neutrophil (%) | 89.9 (82.45, 96) | 89 (84.28, 91.18) | 0.627 |
| Platelet (×10^9^/L) | 109 (55.5, 256) | 122 (29, 215.25) | 0.471 |
| ALT (U/L) | 24 (11.5, 58) | 36 (17, 74.75) | 0.576 |
| AST (U/L) | 46 (32.5, 119.5) | 60.5 (21.25, 121.75) | 0.843 |
| Total bilirubin(μmol/L) | 21.48 (9.78, 24.66) | 13.15 (7.58, 27.98) | 0.397 |
| DBil (µmol/L) | 8.5 (4.45, 15.25) | 8.45 (4.18, 21.23) | 0.971 |
| Serum albumin (g/L) | 35.1 (27.45, 40.7) | 27.75 (24.43, 32.18) | **0.040** |
| D-dimer (mg/L) | 2.92 (0.69, 5.21) | 1.94 (1.46, 3.94) | 0.719 |
| Prothrombin time (s) | 13.1 (12.75, 14.5) | 13.05 (11.8, 14.05) | 0.387 |
| CRP (mg/L) | 87.63 (32.14, 167.88) | 155.86 (72.24, 234.22) | 0.117 |
| PCT (ng/mL) | 3.25 (0.67, 13.4) | 3.64 (0.37, 19.87) | 0.885 |
| Urea nitrogen (mmol/L) | 15.9 (12.95, 20.63) | 9.7 (4.28, 16.5) | **0.040** |
| Creatinine (µmol/L) | 90 (56, 122.5) | 95.5 (57.75, 173.75) | 0.773 |
| Mortality (28 days), n (%) | 9 (52.94) | 9 (56.25) | 0.849 |
| Mortality (hospitalization) | 0 (0) | 2 (12.5) | 0.133 |
| Mortality (90 days) | 12 (70.59) | 14 (87.5) | 0.235 |
| Duration of ICU stay (days) | 22 (13.5, 31) | 13.5 (9.5, 25.5) | 0.176 |
| Total hospitalization time (d) | 30 (18, 41) | 15.5 (8.25, 26) | 0.087 |
| Mechanical ventilation time (h) | 202 (18.5, 466.5) | 228 (165, 363) | 0.449 |
| Vasoactive drug use time (h) | 75.7 (18, 202) | 61.5 (0, 122) | 0.394 |
| Total hospitalization cost (CNY) | 427682 (242268.55, 495129.7) | 240367.99 (161404.96, 429387.5) | 0.130 |

**Supplementary Table 3. Comparison of antimicrobial adjustment for outcomes according to mNGS results or not.**

| Characteristics | Patients received antimicrobial adjustment (n=101) | According to mNGS result (n=45) | Not according to mNGS result (n=56) | *P* value^*^ |
| --- | --- | --- | --- | --- |
| Gender, male, n (%) | 65 (64.4) | 29 (64.4) | 36 (64.3) | 0.987 |
| Age, years | 54 (44, 67) | 52 (43.5, 70) | 55 (44.5, 65.8) | 0.886 |
| Mortality (28 days), n (%) | 51 (50.5) | 22 (48.9) | 29 (51.8) | 0.772 |
| Mortality (hospitalization) | 5 (5.0) | 3 (6.7) | 2 (3.6) | 0.802 |
| Mortality (90 days) | 64 (63.4) | 30 (66.7) | 34 (60.7) | 0.537 |
| Duration of ICU stay (days) | 13 (7, 26) | 13 (4, 25) | 14 (7, 27.5) | 0.211 |
| Total hospitalization time (d) | 22 (10, 40.5) | 22 (10, 43.5) | 21.5 (10.25, 37) | 0.926 |
| Mechanical ventilation time (h) | 100 (0, 264) | 144 (6.5, 299.5) | 77 (0, 231) | 0.341 |
| Vasoactive drug use time (h) | 50 (0, 148) | 50 (12.5, 183.5) | 55.5 (0, 147) | 0.397 |
| Total hospitalization cost (CNY) | 235859(143860.67, 431889) | 203141(90147, 447556) | 249167 (159842, 429756) | 0.436 |

*P* value^*^: according to mNGS vs not according to mNGS result
